# Birth Weight and Risk of Adiposity among Adult Inuit in Greenland

**DOI:** 10.1371/journal.pone.0115976

**Published:** 2014-12-31

**Authors:** Pernille Falberg Rønn, Lærke Steenberg Smith, Gregers Stig Andersen, Bendix Carstensen, Peter Bjerregaard, Marit Eika Jørgensen

**Affiliations:** 1 Clinical Epidemiology, Steno Diabetes Center A/S, Gentofte, Denmark; 2 Centre for Health Research in Greenland, National Institute of Public Health, University of Southern Denmark, Copenhagen, Denmark; University of Leipzig, Germany

## Abstract

**Objective:**

The Inuit population in Greenland has undergone rapid socioeconomic and nutritional changes simultaneously with an increasing prevalence of obesity. Therefore, the objective was to examine fetal programming as part of the aetiology of obesity among Inuit in Greenland by investigating the association between birth weight and measures of body composition and fat distribution in adulthood.

**Methods:**

The study was based on cross-sectional data from a total of 1,473 adults aged 18–61 years in two population-based surveys conducted in Greenland between 1999–2001 and 2005–2010. Information on birth weight was collected from birth records. Adiposity was assessed by anthropometry, fat mass index (FMI), fat-free mass index (FFMI), and visceral (VAT) and subcutaneous adipose tissue (SAT) estimated by ultrasound. The associations to birth weight were analyzed using linear regression models and quadratic splines. Analyses were stratified by sex, and adjusted for age, birthplace, ancestry and family history of obesity.

**Results:**

Spline analyses showed linear relations between birth weight and adult adiposity. In multiple regression analyses, birth weight was positively associated with BMI, waist circumference, FMI, FFMI and SAT with generally weaker associations among women compared to men. Birth weight was only associated with VAT after additional adjustment for waist circumference and appeared to be specific and inverse for men only.

**Conclusions:**

Higher birth weight among Inuit was associated with adiposity in adulthood. More studies are needed to explore a potential inverse association between birth size and VAT.

## Introduction

The Inuit population in Greenland has undergone rapid social, nutritional and health transitions during the last decades, coupled with high levels of obesity, type 2 diabetes and ischemic heart disease [1], [2].

Globally, changes in lifestyle behaviors as a result of transition and urbanization patterns may play an important role in explaining the observed increase in obesity prevalence and related co-morbidities [3]. However, fetal growth has also been proposed to contribute to the development of later chronic disease [4], [5] through a mismatch between intrauterine and adult life environments as suggested by the fetal origins hypothesis [6]. Birth weight has been widely studied as a proxy of fetal growth, and especially low birth weight has been linked to intrauterine growth restriction (IUGR) and adverse health outcomes, particularly type 2 diabetes [7], [8]. Also a link between birth weight and subsequent obesity has been suggested, but the relevant mechanisms in humans are still unclear. Studies are not consistent in their findings with both linear, J- and U-shaped relations [4], though a positive linear relation between birth weight and obesity dominate in most studies [9], [10]. It is therefore paradoxical that studies consistently show a negative association between birth weight and type 2 diabetes [4], [7], [11]. It could be hypothized that these diverging findings are caused by an association between birth weight and accumulation of visceral abdominal tissue (VAT) rather than overall obesity. Less is known about the relation between birth weight and accumulation of VAT and subcutaneous abdominal tissue (SAT) in adult life. A better understanding of the development of these tissues is important because excessive VAT has been shown to be strongly associated with metabolic disturbances and related to insulin resistance [12], whereas SAT may have independent anti-atherogenic effects and other protective properties [13].

The role of birth weight in the development of obesity has to our knowledge never been studied in Greenland or in other Arctic populations. The aim of our study was therefore to investigate the relations between birth weight and adult body composition and fat distribution measured by ultrasonography among the adult Inuit population in Greenland.

## Materials and Methods

### Study population

The study was based on cross-sectional data from two population-based surveys conducted in Greenland in 1999–2001 and 2005–2010. Participants were selected as a random sample of adult Inuit in Greenland (aged 18+ years). For both studies, participants were recruited to fill out self-administered questionnaires and personal interviews, and participated in a clinical examination. Written informed consent was obtained from participants before investigation, and the studies were approved by the ethical review committee of Greenland, and have thus been performed in accordance with the 1964 Declaration of Helsinki and its later amendments. The study methods are described in detail elsewhere [14], [15].

From a total of 4,221 participants who went through clinical examinations, information on birth weight was available for 1,532 ethnic Greenlanders, primarily those born after 1950. Of those with available birth weight information we excluded individuals born before 1950, as birth weight before this year was not systematically registered (n = 6). Moreover, 50 individuals were excluded due to missing values on one or more of selected covariates. The remaining 1,473 individuals constituted the study population of the present study.

### Measurements

Information on birth weight (kg) was gathered from medical records at hospitals. The records included birth records, midwife records and outpatient records of participants, and for the latest years information from the central birth register of Greenland. There was no systematic difference in covariates between individuals with traced and non-traced birth records except for place of birth (town vs. village); slightly more individuals in towns had available information on birth weight compared to individuals in villages. The questionnaires used as survey instruments for personal interviews and self-administered questionnaires were available in both Greenlandic and Danish. A trained research team performed all clinical measurements.

#### Anthropometric measures

Weight (kg) and height (cm) were measured with the participants wearing underwear and socks. Waist circumference (cm) was measured midway between the rib cage and the iliac crest on the standing participant. BMI was calculated as weight divided by height squared (kg/m^2^).

#### Ultrasonography

Measures of intra-abdominal tissue divided into SAT and VAT were assessed by ultrasonography according to a validated protocol [16], [17] performed by a portable ultrasound scanner (Pie Medical) using a 3.5 MHz transducer with the participant in supine position and at the end of a normal expiration. The distances between the posterior edge of the abdominal muscles and the lumbar spine was measured using electronic calipers. Both measurements were obtained from where the xiphoid line and the waist circumference met. Distances were measured from three different angles: medial, 10 cm left and 10 cm right lateral. VAT was defined as the depth (cm) from the peritoneum to the lumbar spine and SAT (cm) was defined as the depth from the skin to the linea alba [14].

#### Bio impedance

Bio impedance and calculation of fat mass were performed on a leg-to-leg Tanita TBF-300MA (Tanita Corporation, Tokyo, Japan). Fat mass was calculated as the total weight of fat from the internal algorithm of the device based on height, weight, sex, impedance and age [14]. Fat-free mass (kg) was calculated as the total body mass minus fat mass. FMI (kg/m^2^) was calculated as fat mass divided by height squared and FFMI (kg/m^2^) was calculated as fat-free mass divided by height squared.

#### Additional covariates

Information on sex and age was retrieved from the central personal register, while birthplace, ancestry, and family history of obesity were collected from the survey questionnaires. Birthplace was categorized as either town or village. Ancestry was based on questions on ethnicity of the four grandparents, and if this information was missing, on the parents' ethnicity. The variable was subsequently recoded as full Inuit (all grandparents were Greenlanders) and mixed Inuit (at least one grandparent or parent was not a Greenlander). Family history of obesity was determined based on whether the participant's parents and/or siblings were reported obese or severely overweight. The variable was dichotomized as yes (any parent or sibling was regarded obese/overweight) and no.

### Statistical analyses

Regression models were used to assess the effect estimates of associations between birth weight and adiposity measures. To obtain symmetry of residuals, the adiposity measures were all log-transformed; coefficients were therefore backtransformed and expressed as percentage change per 1 kg change in birth weight. In all models we tested for a sex-birth weight interaction, which resulted in significant interactions in models with waist circumference and FFMI. All analyses were, however, stratified on sex due to biological differences in body composition and fat distribution between genders. The linear regression analyses were performed in three steps; unadjusted regression analyses of the adiposity measures as a function of birth weight (**model 1**); multiple regression analyses with adjustment for the chosen potential confounders (age, birthplace, ancestry and family history of obesity) (**model 2**); and regression analyses with further adjustment for adult waist circumference in models including VAT and SAT as outcome measures (**model 3**). Model 3 was performed to examine the effect of birth weight on VAT and SAT in adulthood conditioned on the effect of adult abdominal body size.

We used multiple regression with quadratic splines based on model 2 to visually investigate the relations between birth weight (explanatory variable) and measures of adult adiposity (BMI, waist circumference, FMI, FFMI, VAT and SAT) (outome variables). The splines were interpolated with three knots placed at 2.5 kg, 3.5 kg and 4.5 kg on the birth weight scale. We calculated predicted values for a person with prespecified values of the covariates (33 years, full Inuit, born in a town and with a family history of obesity). We tested for linearity by comparing the spline model to a model with linear effect of birth weight.

Statistical analyses were performed using the statistical software SAS, version 9.3 (SAS Institute Inc., Carey, NC, USA) and R, version 3.0.2 [18].

## Results

### Characteristics of the study population

Population characteristics are summarized in **Table 1**. The mean age of participants was 33 years (range 18-61). Men had a greater mean birth weight than women (3415±531 g vs. 3277±569 g) and a total of 89 participants (6%) had a low birth weight defined as a weight below 2500 g. Obesity regarded as a BMI≥30 kg/m^2^ was reported among 15% of men and 19% of women. Women had a higher mean fat mass and mean SAT than men, while men on average had a higher fat-free mass and VAT than women.

**Table 1:** Characteristics of the study population (n = 1473).

		Men (n = 628)	Women (n = 845)
Age		33.6±9.1^a^	33.1±8.8
Birth weight (g)		3415±531	3277±569
Birth length (cm)		51.7±2.3	50.7±2.5
Weight (kg)		74.0±14.2	66.4±14.5
Height (cm)		171.5±6.5	159.3±6.2
BMI (kg/m^2^)		25.1±4.2	26.1±5.3
Waist circumference (cm)		88.9±12.1	88.3±13.4
Fat mass (kg)		15.3±8.8	22.7±11.0
Fat mass index (kg/m^2^)		5.2±2.9	8.9±4.2
Fat-free mass (kg)		58.2±6.9	43.9±4.7
Fat-free mass index (kg/m^2^)		19.9±1.7	17.4±1.6
Visceral adipose tissue (cm)		7.1±2.1	6.3±2.0
Subcutaneous adipose tissue (cm)		2.2±1.3	3.7±1.5
Birthplace			
	Town	459 (73.1)^b^	616 (72.9)
	Village	169 (26.9)	229 (27.1)
Ancestry			
	Full Inuit	529 (84.2)	716 (84.7)
	Mixed Inuit	99 (15.8)	129 (15.3)
Family history of obesity			
	No	508 (80.9)	618 (73.1)
	Yes	120 (19.1)	227 (26.9)

a Mean ± SD (all such values).

b n (%) (all such values).

### Shape of the associations

**Figure 1:**
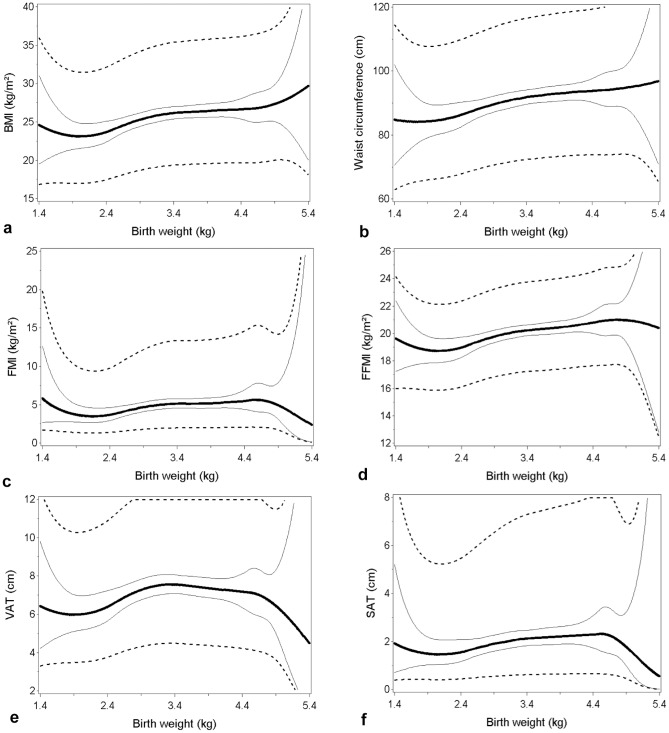
Splines showing the relations between birth weight and measure of adult adiposity for men. Quadratic splines of the relation between birth weight and (a) body mass index (BMI), (b) waist circumference, (c) fat mass index (FMI), (d) fat-free mass index (FFMI), (e) visceral adipose tissue (VAT) and (f) subcutaneous adipose tissue (SAT). The thick lines represent the relations are predicted for a person aged 33, being full Inuit, born in a town, and reported family history of obesity. The full thin lines show the 95% CI and dotted lines show the 95% prediction interval.

**Figure 2:**
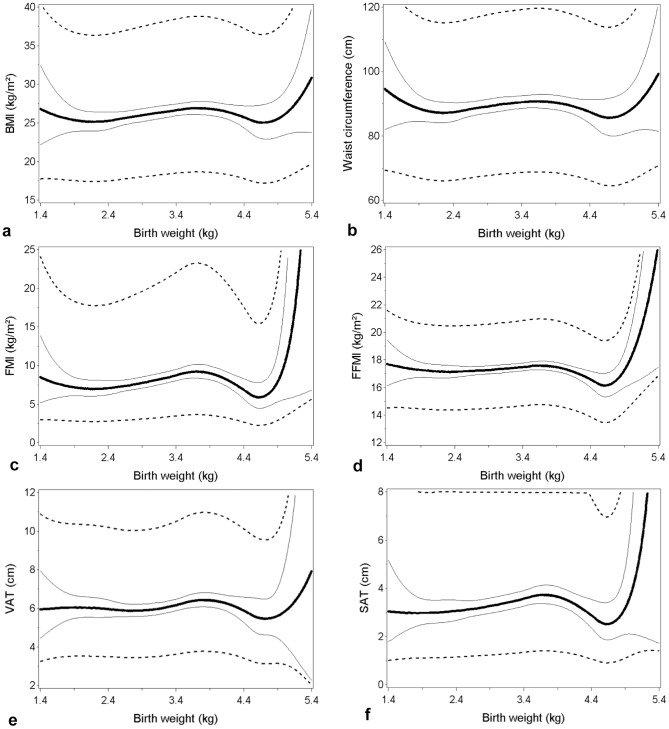
Splines showing the relations between birth weight and measures of adult adiposity for women. Quadratic splines of the relation between birth weight and (a) body mass index (BMI), (b) waist circumference, (c) fat mass index (FMI), (d) fat-free mass index (FFMI), (e) visceral adipose tissue (VAT) and (f) subcutaneous adipose tissue (SAT). The thick lines represent the relations predicted for a person aged 33, being full Inuit, born in a town, and reported family history of obesity. The full thin lines show the 95% CI and dotted lines show the 95% prediction interval.

**Fig. 1** **(a-f)** and **Fig. 2** **(a-f)** show the shape of the associations for the continuous relations between birth weight and the different adiposity outcomes for men and women, respectively, estimated by using quadratic splines. The spline analyses in general showed no indication of non-linear associations. Neither did the tests for linearity indicate deviation from linearity except in models with FMI (*p* = 0.006) and FFMI (*p* = 0.025) as adiposity outcome among women, where there was an indication of an increase for a birth weight above 4.5 kg.

### Birth weight and body composition

**Figure 3:**
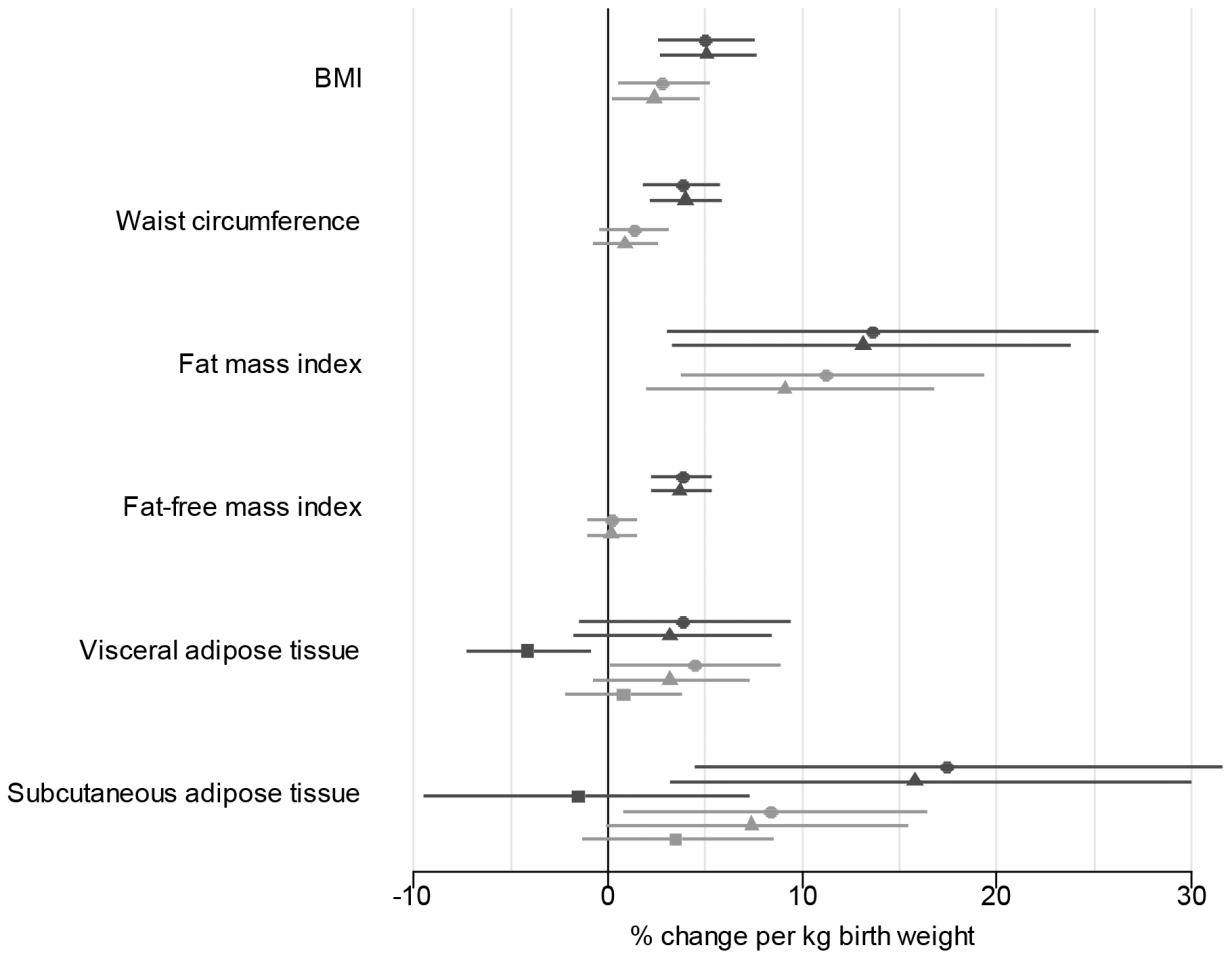
Linear associations between birth weight and measures of adult adiposity. Estimates for men (black) and women (grey) with 95% CI. • Model 1: Unadjusted. ▴ Model 2: Adjusted for age, birthplace, ancestry and family history of obesity. ▪ Model 3: Adjusted for as model 2 incl. waist circumference.

The results of the multiple linear regression models are shown in **Table 2** and the estimates with 95% CI are plotted in **Fig. 3** **.** Birth weight was positively associated with BMI and FMI for both genders after adjustment for potential confounders (model 2). A difference in birth weight of 1 kg corresponded to a 13.1% (95% CI: 3.3; 23.8) higher FMI for men, and a 9.1% (95% CI: 2.0; 16.8) higher FMI for women. Moreover, there was a significant positive association between birth weight and FFMI among men only with a 3.7% (95% CI: 2.2; 5.3) higher FFMI per kg difference in birth weight, significantly different from that in women.

**Table 2:** Associations between birth weight and measures of adiposity stratified by sex.

		% Increment per kg increment in birth weight (95% CI)			% Increment per cm increment in waist circumference (95% CI)
Outcome variables		Model 1^a^	Model 2^b^	Model 3^c^^,^ d	
Men					
	BMI	5.0 (2.6, 7.5)	5.1 (2.7, 7.6)		
	Waist circumference	3.8 (1.8, 5.8)	4.0 (2.1, 5.9)		
	Fat mass index	13.6 (3.0, 25.2)	13.1 (3.3, 23.8)		
	Fat-free mass index	3.8 (2.2, 5.3)	3.7 (2.2, 5.3)		
	Visceral adipose tissue	3.8 (−1.5, 9.4)	3.2 (−1.8, 8.4)	−4.1 (−7.3, −0.9)	1.2 (1.6,1.9)
	Subcutaneous adipose tissue	17.4 (4.4, 32.1)	15.8 (3.2, 30.0)	−1.5 (−9.5, 7.3)	3.9 (3.5, 4.3)
Women					
	BMI	2.8 (0.5, 5.2)	2.4 (0.2, 4.7)		
	Waist circumference	1.3 (−0.4, 3.1)	0.9 (−0.8, 2.6)		
	Fat mass index	11.2 (3.7, 19.3)	9.1 (2.0, 16.8)		
	Fat-free mass index	0.2 (−1.1, 1.5)	0.2 (−1.1, 1.5)		
	Visceral adipose tissue	4.4 (0.1, 8.9)	3.2 (−0.8, 7.3)	0.8 (−2.2, 3.8)	1.4 (1.2, 1.5)
	Subcutaneous adipose tissue	8.3 (0.8, 16.4)	7.4 (−0.1, 15.4)	3.5 (−1.3, 8.5)	2.8 (2.7, 3.1)

a Model 1. Unadjusted model.

b Model 2. Adjusted for age, birthplace, ancestry and family history of obesity.

c Model 3. Adjusted for age, birthplace, ancestry, family history of obesity and waist circumference (left column).

d Model 3. Adjusted for age, birthplace, ancestry, family history of obesity and birth weight (right column).

### Birth weight and fat distribution

Birth weight was positively related to waist circumference indicating an increased risk of overall abdominal obesity. There was, moreover, a positive association between birth weight and SAT among men with a 15.8% (95% CI: 3.2; 30.1) higher SAT per kg birth weight (model 2), and the same but weaker trend among women. Birth weight was not associated with VAT, however, additional adjustment for waist circumference (model 3) resulted in an inverse association with VAT among men only, with a decrease of 4.1% (95% CI: 0.9; 7.3) per kg difference in birth weight conditioned on waist circumference.

## Discussion

We aimed to investigate the relations between birth weight and adult body composition and fat distribution among the Inuit population in Greenland. Our findings showed that birth weight was positively associated with BMI, waist circumference, FMI and FFMI with generally weaker associations among women as compared to men. Birth weight was similarly positively associated with SAT, whereas an association with VAT was only apparent after adjustment for adult waist circumference for men.

### Body composition

The demonstrated relation between birth weight and FMI emphasizes that higher birth weight increases the relative fat mass in adulthood. Furthermore, we also found that birth weight was more strongly associated with FMI than with FFMI. This finding contradicts results from other studies reporting positive associations between birth weight and fat-free mass rather than fat mass [19]–[21]. Evidence on the relation between birth weight and fat mass is, however, not clear.

A challenge mentioned in the literature is that absolute measures of lean and fat mass represent, not only the proportion of compartments, but also body size itself [22]. We addressed this problem by including indexes of fat and fat-free mass in analyses, which are considered as more appropriate measures because they are adjusted for variability in height [23].

To our knowledge, this is the first study conducted in Greenland or in any other Arctic population, examining birth weight and later development of adult adiposity. An explanation of the observed associations between birth weight and measures of body composition is that the impact of fetal growth on the tendency to deposit fat relative to lean mass is specific for Inuit. An association between the thermal environment during pregnancy and body mass in the offspring has been suggested in the literature, pointing at climatic adaptions which might already start during fetal life [24]. Hence, mechanisms of thermogenesis in body weight regulation could partly explain the relation between birth weight and relative composition of adult fat mass and fat-free mass in Inuit found in the present study. Consequently, it might be problematic to generalize effects of birth weight on later body composition between populations due to ethnic differences in tissue development in fetal life.

### Fat distribution

When adjusting for waist circumference we found a statistically significant inverse association between birth weight and VAT among men. There was no statistically significant effect among women, but the difference in effects between men and women were not significant either, suggesting that the association was spurious.

The adjustment for waist circumference in our study was biologically and clinically grounded, as we wanted to elucidate the independent effect of birth weight on VAT and SAT by removing the impact of individual abdominal body size. Other studies have similarly adjusted for an overall adiposity measure, most frequently BMI [25]–[27]. Replacing waist circumference with BMI in our analyses showed the same results (results not shown). Rolfe *et al* found a similar relation for men and women together after adjustment for overall adiposity [25]. In contrast, studies by McNeely *et al* [26] and Choi *et al* [28] found no associations between birth weight and VAT. Small sample sizes in these studies could have underpowered their ability to find associations. A cohort study by Demerath *et al* [29] measuring VAT and SAT by MRI in adults suggested that infant weight gain, and not birth weight, was positively associated with adult VAT. In comparison with these studies, our finding of an inverse association between birth weight and VAT among Inuit men was based on a large sample of adults, giving more statistical power to the analyses. The inverse association in our model could be interpreted as if low birth weight is associated with a relatively higher amount of VAT in adulthood. However, it has been shown that in analyses where birth weight is adjusted for a measure of adult body size, it is not possible to disentangle the effects of body size at birth and the effect of change in body size between birth and adulthood [30]. In fact, Lucas and Cole argued, that a number of previous studies assessing adult effects of birth body size, were biased due to this so called “reversal paradox”. This is a statistical phenomenon introduced when a relation between birth body size and adult health outcomes is adjusted for adult body size. When such adjustment is done it commonly results in a reversal of the relation between body size at birth and adult health outcomes, which may mistakenly be interpreted as a real inverse relation between birth body size and adult outcome [31]. Ideally, repeated measurements of body size through the life course should be used to explain to which extent and at which time points change in body size affects adult VAT, but in the present study we did not have repeated measurements of body size available. Therefore it is unknown to which extent the inverse relation between birth weight and VAT is attributable to an effect of change in body size somewhere between birth and adult life.

We have followed the recommendation of Lucas and Cole [30] and included also the analyses adjusted for adult waist circumference. The effect of waist circumference on VAT and SAT independent of birth weight and other confounders show a positive association between waist circumference and VAT and SAT, which is not surprising, since both VAT and SAT contribute to waist circumference. In the models with waist circumference as additional explanatory variable, the effect of birth weight on VAT and SAT must be interpreted as effects for a given waist circumference; that is on the relative size of VAT. This relationship between birth weight and VAT for a given adult waist circumference could naturally be further explained by an interaction allowing the birth weight effect on VAT to be different for different levels of waist circumference. However, our data material was not sufficiently large for this type of detailed analyses to be conducted.

Our findings of positive associations between birth weight and SAT are in contrast to the findings by Rolfe *et al*, where birth weight was suggested to have limited influence on SAT. A recent study found that the level of SAT among the Inuit population in general is higher compared to other populations, while VAT does not differ from other populations [32]. Following a hypothesis of evolutionary adaptions to climate regulating thermogenesis [24], mechanisms possibly influence adaptions in Inuit in terms of thicker subcutaneous layers, making them more likely to distribute fat subcutaneously because of cold temperatures. Furthermore, it has been suggested that higher birth weight protects against stresses during early life like hypothermia in colder environments by incorporating greater levels of energy stores [24], [33], and that subcutaneous abdominal fat correlates favorably with health and disease risk in individuals [34].

Except for the association between birth weight and VAT among men conditional on waist circumference, our results did not show an increase in adiposity among individuals with low birth weight. These findings are in agreement with results from a meta-analysis by Schellong *et al* [10] suggesting no indication of U-shaped associations between birth weight and adult overweight. However, our analyses used log-transformed measures so when transformed back to the original log-scale, linear relationships were curved (convex).

Overall, our results indicate that birth weight influences later development of adult adiposity among Inuit in Greenland. Whether our results are due to fetal programming and support the fetal origins hypothesis is hard to determine. Greenland has like many other countries undergone a rapid transition towards a more westernized lifestyle [2], but this pattern might be specific for Greenland and not necessarily comparable with changes seen in low income countries, where the fetal origins hypothesis in particular has been confirmed. Despite quite consistent evidence for fetal programming as a phenomenon playing a role for later health across populations [10], the Inuit seem to be protected against some diseases compared to other populations, e.g. Greenlanders have lower blood pressure and lower cholesterol levels for a given BMI compared to Danes [35]. These differences in risk profiles and disease patterns could indicate ethnic-specific pathways by which development of disease is programmed.

A major strength of this study is that information on birth weight is collected from birth records in contrast to many previous studies that have used self-reported birth weight, which could have introduced recall bias. The large number of participants with fat distribution measured by ultrasound is another strength of this study. The relation between birth size and intra-abdominal fat distribution measured by ultrasound has to our knowledge only previously been studied by Rolfe *et al* examining 1,092 adults in the UK [25].

The use of splines to examine the shape of the associations made it possible to explore and test non-linear relations between birth weight and adult adiposity, and to establish that the log-transformed adiposity measures approximated linear associations with birth weight. The effect of log-transformation, and the results expressed in percentage change (see Table 2), makes interpretation of the relatively small differences in adiposity mean values across the scale of birth weight simple and comprehensible. While studies consistently have found low birth weight to be associated with increased risk of type 2 diabetes [7], [11], evidence on the shape of the association between birth weight and adult adiposity is unclear [10], [36]. Our findings overall suggest a (log-)linear relation between low birth weight and adiposity except with VAT adjusted for waist circumference.

As a consequence of lack of information on gestational age we were not able to differentiate between infants born preterm and IUGR infants. Gestational age could therefore confound our findings. However, a number of studies adjusting for gestational age, suggest that gestational age only has limited impact on the association between birth size and later adiposity [10], [20], [22]. Moreover, the use of birth weight as a proxy of fetal growth can be problematic, for instance maternal nutrition does not equal fetal nutrition [37]. Studies with more accurate measures particularly of gestational age to detect fetal growth could help elucidate the relation with IUGR. Maternal smoking could similarly be an unmeasured confounder in our study. The smoking prevalence in Greenland is high [38] and maternal smoking in Inuit women has been significantly associated with low birth weight [39], [40]. Studies have additionally found increased BMI in adults of mothers who smoked during pregnancy [41].

In conclusion, our data suggest that higher birth weight is associated with a higher degree of adiposity in adulthood. In men, low birth weight is associated with an increased risk for accumulation of VAT after adjustment for waist circumference. However, in the present study we cannot determine whether this result is an effect of birth weight or a result of change in body size between birth and adulthood. Longitudinal studies with repeated measurements of adiposity are needed to distinguish the impact of postnatal growth from that of birth size.
